# Supplementary material for: Mendelian randomization analyses explore the relationship between cathepsins and lung cancer
Source: Commun Biol. 2023 Oct 7;6:1019. doi: 10.1038/s42003-023-05408-7 (PMC10560205; doi:10.1038/s42003-023-05408-7)
Supplement: Supplementary file 2 — Supplementary Information [file 42003_2023_5408_MOESM2_ESM.pdf]

Supplementary Table 1. Pleiotropy and heterogeneity analyses of Two-Sample MR between cathepsins family and different histological typ

exposure	outcome	MR-Egger intercept		MR-PRESSO global	MR-IVW			MR-Egeer		
		Egger intercept	p_value	p_value	Q	Q_df	Q_pval	Q	Q_df	Q_pval
cathepisn B	Overall lung cancer	-0.002	0.619	0.841	10.268	15	0.803	10.244	14	0.744
	Adenocarcinoma	-0.019	0.278	0.595	12.638	14	0. 555	13.915	15	0.532
	Squamous carcinoma	0.004	0.835	0.645	11.318	13	0.584	11.273	12	0.506
	SCLC	0.059	0.261	0.084	20.612	11	0.038	18.054	10	0.054
cathepisn E	Overall lung cancer	-0.004	0.751	0.820	5.254	9	0.812	5.146	8	0.742
	Adenocarcinoma	-0.016	0.376	0.586	7.264	9	0.610	6.386	8	0.604
	Squamous carcinoma	-0.012	0.658	0.473	7.815	8	0.452	7.583	7	0.371
	SCLC	-0.037	0.299	0.299	12.465	9	0.188	10.797	8	0.213
cathepisn F	Overall lung cancer	-0.014	0.320	0.515	9.209	10	0.512	8.100	9	0.524
	Adenocarcinoma	-0.026	0.284	0.173	15.529	10	0.114	13.573	9	0.138
	Squamous carcinoma	-0.005	0.816	0.701	7.676	10	0.660	7.619	9	0.573
	SCLC	-0.041	0.264	0.667	7.502	10	0.677	6.086	9	0.731
cathepisn G	Overall lung cancer	-0.001	0.967	0.309	12.134	10	0.276	12.131	9	0.206
	Adenocarcinoma	-0.019	0.366	0.750	6.742	10	0.750	5.837	9	0.756
	Squamous carcinoma	0.003	0.908	0.399	9.894	9	0.359	9.876	8	0.274
	SCLC	0.021	0.674	0.074	17.291	10	0.068	16.936	9	0.050
cathepisn H	Overall lung cancer	-0.004	0.702	0.270	15.432	9	0.080	15.134	8	0.057
	Adenocarcinoma	-0.007	0.603	0.433	10.420	9	0.318	10.052	8	0.261
	Squamous carcinoma	0.010	0.538	0.347	13.054	10	0.221	12.485	9	0.187
	SCLC	0.021	0.421	0.430	7.676	8	0.466	6.945	7	0.435
cathepisn L2	Overall lung cancer	-0.022	0.169	0.156	15.009	10	0.132	12.026	9	0.212
	Adenocarcinoma	-0.010	0.630	0.452	9.263	9	0.413	8.982	8	0.344
	Squamous carcinoma	0.003	0.931	0.072	17.830	10	0.058	17.814	9	0.037
	SCLC	-0.015	0.663	0.884	3.349	8	0.911	3.143	7	0.871
cathepisn O	Overall lung cancer	0.016	0.227	0.799	6.806	10	0.744	5.127	9	0.823
	Adenocarcinoma	0.023	0.215	0.871	5.882	10	0.825	4.105	9	0.904
	Squamous carcinoma	-0.001	0.975	0.855	6.322	10	0.788	6.321	9	0.707
	SCLC	0.013	0.804	0.212	8.970	7	0.255	8.871	6	0.181
cathepisn S	Overall lung cancer	0.019	0.022	0.074	30.317	19	0.048	22.495	18	0.211
	Adenocarcinoma	0.018	0.066	0.211	24.987	20	0.202	20.805	19	0.348
	Squamous carcinoma	0.023	0.049	0.134	29.214	22	0.139	24.204	21	0.283
	SCLC	0.025	0.141	0.777	14.462	19	0.756	12.087	18	0.843
cathepisn Z	Overall lung cancer	0.025	0.141	0.757	14.462	19	0.756	12.087	18	0.843
	Adenocarcinoma	0.025	0.141	0.763	14.462	19	0.756	12.087	18	0.843
	Squamous carcinoma	0.025	0.141	0.766	14.462	19	0.756	12.087	18	0.843
	SCLC	0.025	0.141	0.760	14.462	19	0.756	12.087	18	0.843

Supplementary Table 2. The results of reverse MR analysis of different histological of lung cancer and cathepsins family

exposure	outcome	nsnp	Inverse variance weighted			MR-Egger			Weighted median			MR-Egger intercept		MR-PRESSO global	MR-IVW			MR-Egeer		
			p_value	OR	95% OR	p_value	OR	95% OR	p_value	OR	95% OR	Egger intercept	p_value	p_value	Q	Q_df	Q_pval	Q	Q_df	Q_pval
	Cathepsin B	15	0.352	1.074	0.924-1.278	0.312	1.255	0.822-1.916	0.373	1.101	0.891-1.361	-0.018	0.454	0.644	11.340	14	0.659	10.743	13	0.632
	Cathepsin E	15	0.502	1.058	0.897-1.247	0.600	1.141	0.705-1.846	0.554	1.070	0.860-1.337	-0.009	0.747	0.262	16.959	14	0.258	16.819	13	0.208
	Cathepsin F	15	0.504	0.948	0.811-1.108	0.554	0.868	0.551-1.369	0.367	0.907	0.731-1.122	0.010	0.692	0.360	15.243	14	0.362	15.053	13	0.304
	Cathepsin G	15	0.747	1.025	0.880-1.194	0.427	1.201	0.775-1.863	0.710	1.039	0.848-1.272	-0.018	0.463	0.443	14.572	14	0.408	13.957	13	0.377
Overall lung cancer	Cathepsin H	15	0.447	1.107	0.852-1.439	0.789	1.114	0.515-2.408	0.953	1.006	0.813-1.245	-0.001	0.988	0.063	43.145	14	0.000	43.144	13	0.000
	Cathepsin L2	15	0.557	0.955	0.819-1.113	0.233	0.760	0.494-1.169	0.873	0.983	0.802-1.210	0.026	0.286	0.415	14.744	14	0.396	13.462	13	0.413
	Cathepsin O	15	0.226	1.110	0.937-1.316	0.326	1.291	0.790-2.108	0.460	1.082	0.877-1.336	-0.017	0.532	0.236	18.003	14	0.207	17.449	13	0.180
	Cathepsin S	15	0.325	1.078	0.928-1.252	0.520	0.867	0.568-1.324	0.422	1.082	0.892-1.313	0.025	0.301	0.981	5.112	14	0.984	3.950	13	0.992
	Cathepsin Z	15	0.191	1.117	0.947-1.317	0.365	0.807	0.516-1.263	0.284	1.121	0.909-1.383	0.037	0.152	0.255	17.102	14	0.251	14.521	13	0.338
	Cathepsin B	14	0.763	0.979	0.854-1.123	0.536	1.169	0.724-1.888	0.322	0.914	0.765-1.092	-0.024	0.464	0.243	16.042	13	0.247	15.312	12	0.225
	Cathepsin E	14	0.255	1.074	0.950-1.215	0.496	1.164	0.761-1.780	0.150	1.131	0.956-1.338	-0.011	0.705	0.617	10.781	13	0.629	10.631	12	0.561
	Cathepsin F	14	0.495	1.049	0.914-1.205	0.967	1.011	0.616-1.659	0.967	1.076	0.908-1.275	0.005	0.880	0.250	16.352	13	0.231	16.319	12	0.177
	Cathepsin G	14	0.595	1.034	0.914-1.170	0.158	1.385	0.906-2.118	0.281	1.089	0.933-1.270	-0.040	0.183	0.712	9.705	13	0.718	7.712	12	0.807
Adenocarcinoma	Cathepsin H	14	0.598	1.062	0.848-1.331	0.476	0.746	0.342-1.627	0.800	0.977	0.817-1.169	0.048	0.372	0.142	43.402	13	0.000	40.497	12	0.000
	Cathepsin L2	14	0.184	0.920	0.813-1.040	0.131	0.704	0.461-1.076	0.579	0.954	0.806-1.128	0.037	0.221	0.847	7.529	13	0.873	5.863	12	0.923
	Cathepsin O	14	0.731	0.979	0.865-1.107	0.117	0.693	0.454-1.060	0.970	1.003	0.843-1.195	0.047	0.122	0.417	12.171	13	0.514	9.406	12	0.668
	Cathepsin S	14	0.466	1.051	0.920-1.200	0.691	1.103	0.687-1.773	0.487	1.059	0.901-1.245	-0.007	0.836	0.327	12.171	13	0.514	9.406	12	0.668
	Cathepsin Z	14	0.328	1.078	0.928-1.251	0.260	0.745	0.456-1.215	0.546	1.057	0.884-1.263	0.051	0.148	0.125	12.171	13	0.514	9.406	12	0.668
	Cathepsin B	6	0.033	1.189	1.014-1.395	0.438	1.264	0.741-2.156	0.038	1.224	1.011-1.481	-0.011	0.826	0.804	2.523	5	0.773	2.468	4	0.650
	Cathepsin E	6	0.763	1.032	0.843-1.263	0.325	0.694	0.366-1.315	0.640	1.055	0.842-1.323	0.074	0.272	0.172	8.065	5	0.153	5.738	4	0.220
	Cathepsin F	6	0.437	0.917	0.737-1.141	0.279	0.630	0.306-1.300	0.037	0.789	0.631-0.986	0.070	0.347	0.118	9.431	5	0.093	7.352	4	0.118
	Cathepsin G	6	0.870	1.015	0.846-1.219	0.470	1.296	0.685-2.450	0.967	0.996	0.813-1.220	-0.045	0.476	0.286	6.590	5	0.253	5.708	4	0.222
Squamous carcinoma	Cathepsin H	6	0.758	0.975	0.832-1.144	0.979	0.992	0.582-1.693	0.601	0.950	0.782-1.153	-0.003	0.950	0.982	0.651	5	0.986	0.647	4	0.958
	Cathepsin L2	6	0.146	0.882	0.744-1.045	0.136	0.602	0.353-1.027	0.635	0.950	0.767-1.176	0.071	0.216	0.350	5.664	5	0.340	3.511	4	0.476
	Cathepsin O	6	0.964	1.004	0.842-1.197	0.566	0.820	0.439-1.530	0.979	1.003	0.805-1.250	0.038	0.541	0.307	6.072	5	0.299	5.464	4	0.243
	Cathepsin S	6	0.678	0.967	0.824-1.134	0.640	1.147	0.673-1.956	0.916	1.010	0.834-1.223	-0.032	0.546	0.585	3.935	5	0.559	3.502	4	0.478
	Cathepsin Z	6	0.798	0.970	0.770-1.222	0.908	0.947	0.399-2.250	0.512	0.926	0.736-1.165	0.004	0.957	0.082	10.524	5	0.062	10.515	4	0.033
	Cathepsin B	26	0.641	1.017	0.948-1.091	0.156	1.115	0.964-1.291	0.965	1.002	0.914-1.099	-0.024	0.173	0.293	28.358	25	0.292	26.203	24	0.343
	Cathepsin E	26	0.841	1.007	0.942-1.075	0.516	1.048	0.911-1.205	0.345	1.049	0.950-1.157	-0.010	0.528	0.581	23.060	25	0.574	22.650	24	0.541
	Cathepsin F	26	0.509	1.024	0.954-1.100	0.935	1.006	0.863-1.173	0.457	1.037	0.942-1.141	0.004	0.802	0.254	28.986	25	0.264	28.909	24	0.224
	Cathepsin G	26	0.897	1.004	0.940-1.073	0.778	1.021	0.887-1.174	0.958	0.998	0.909-1.095	-0.004	0.802	0.874	17.361	25	0.868	17.297	24	0.836
SCLC	Cathepsin H	26	0.442	0.973	0.906-1.044	0.761	0.976	0.838-1.138	0.645	0.977	0.886-1.078	-0.001	0.957	0.259	28.798	25	0.272	28.795	24	0.228
	Cathepsin L2	26	0.148	1.050	0.983-1.122	0.517	1.048	0.911-1.205	0.470	1.033	0.946-1.127	0.000	0.977	0.920	15.533	25	0.928	15.532	24	0.904
	Cathepsin O	26	0.367	1.031	0.965-1.101	0.890	1.010	0.878-1.162	0.408	1.038	0.950-1.136	0.005	0.749	0.919	16.098	25	0.912	15.993	24	0.888
	Cathepsin S	26	0.464	0.972	0.901-1.049	0.253	0.908	0.772-1.067	0.608	0.976	0.891-1.070	0.018	0.358	0.116	33.419	25	0.121	32.238	24	0.121
	Cathepsin Z	26	0.798	0.970	0.770-1.223	0.908	0.947	0.399-2.250	0.512	0.926	0.736-1.165	0.004	0.957	0.082	10.524	5	0.062	10.515	4	0.033

Supplementary Table 3. The resluts of multivariable MR analysis between cathepsins family and lung cancer risk.

outcome	exposure	SNPs	IVW			MR-egger			MR-IVW			MR-Egeer			MR-Egger intercept	
			pvalue	OR	95% OR	pvalue	OR	95% OR	Q	Q_df	Q_pval	Q	Q_df	Q_pval	Egger intercept	p_value
overall lung cancer	cathepisn B		0.994	1.000	0.957 - 1.045	0.543	1.016	0.966 - 1.068								
	cathepisn E		0.283	1.037	0.970 - 1.109	0.118	1.057	0.986 - 1.133								
	cathepisn F		0.431	1.023	0.966 - 1.084	0.696	1.012	0.954 - 1.073								
	cathepisn G		0.219	1.039	0.977 - 1.105	0.298	1.033	0.972 - 1.098								
	cathepisn H	88	0.000	1.070	1.033 - 1.109	0.000	1.074	1.038 - 1.113	89.810	79.000	0.191	85.904	78.000	0.253	-0.003	0.219
	cathepisn O		0.419	0.967	0.892 - 1.049	0.231	0.951	0.877 - 1.032								
	cathepisn S		0.725	1.007	0.968 - 1.048	0.760	1.006	0.967 - 1.047								
	cathepisn L2		0.924	1.003	0.935 - 1.077	0.623	1.018	0.948 - 1.094								
	cathepisn Z		0.333	0.976	0.928 - 1.025	0.478	0.982	0.935 - 1.032								
Adenocarcinoma	cathepisn B		0.982	1.001	0.944 - 1.060	0.402	1.029	0.963 - 1.099								
	cathepisn E		0.488	1.032	0.944 - 1.127	0.267	1.053	0.961 - 1.155								
	cathepisn F		0.680	1.016	0.941 - 1.097	0.999	1.000	0.926 - 1.080								
	cathepisn G		0.152	1.061	0.978 - 1.151	0.189	1.055	0.974 - 1.144								
	cathepisn H	88	0.000	1.094	1.046 - 1.143	0.000	1.098	1.050 - 1.147	82.350	79.000	0.376	78.705	78.000	0.456	-0.005	0.106
	cathepisn O		0.159	0.926	0.832 - 1.031	0.077	0.908	0.815 - 1.011								
	cathepisn S		0.359	1.025	0.973 - 1.079	0.352	1.024	0.974 - 1.078								
	cathepisn L2		0.513	1.033	0.937 - 1.138	0.317	1.051	0.954 - 1.158								
	cathepisn Z		0.796	0.991	0.927 - 1.060	0.980	1.001	0.937 - 1.070								
Squamous carcinoma	cathepisn B		0.442	1.028	0.958 - 1.103	0.341	1.041	0.958 - 1.131								
	cathepisn E		0.941	0.996	0.888 - 1.116	0.916	1.007	0.891 - 1.137								
	cathepisn F		0.340	1.046	0.954 - 1.147	0.439	1.038	0.944 - 1.142								
	cathepisn G		0.626	1.026	0.926 - 1.136	0.674	1.022	0.922 - 1.134								
	cathepisn H	89	0.175	1.037	0.984 - 1.094	0.157	1.039	0.985 - 1.096	90.022	80.000	0.208	89.465	79.000	0.197	-0.002	0.557
	cathepisn O		0.405	0.947	0.833 - 1.076	0.339	0.938	0.823 - 1.070								
	cathepisn S		0.459	1.023	0.963 - 1.087	0.471	1.023	0.962 - 1.087								
	cathepisn L2		0.830	1.012	0.905 - 1.133	0.712	1.022	0.910 - 1.148								
	cathepisn Z		0.561	0.976	0.901 - 1.058	0.636	0.980	0.903 - 1.064								
SCLC	cathepisn B		0.180	0.924	0.824 - 1.037	0.069	0.888	0.782 - 1.009								
	cathepisn E		0.143	1.131	0.959 - 1.332	0.030	1.200	1.018 - 1.415								
	cathepisn F		0.580	1.042	0.900 - 1.207	0.562	1.044	0.903 - 1.206								
	cathepisn G		0.422	1.066	0.912 - 1.247	0.529	1.049	0.903 - 1.219								
	cathepisn H	83	0.154	1.063	0.978 - 1.155	0.134	1.063	0.981 - 1.152	82.265	74.000	0.239	74.103	73.000	0.442	0.007	0.266
	cathepisn O		0.365	1.102	0.893 - 1.360	0.434	1.085	0.884 - 1.333								
	cathepisn S		0.422	1.041	0.943 - 1.149	0.395	1.042	0.948 - 1.145								
	cathepisn L2		0.990	1.001	0.837 - 1.197	0.937	1.007	0.844 - 1.202								
	cathepisn Z		0.109	0.900	0.791 - 1.024	0.097	0.899	0.794 - 1.019								
