# Supplementary material for: Mendelian randomization analyses explore the relationship between cathepsins and lung cancer
Source: Commun Biol. 2023 Oct 7;6:1019. doi: 10.1038/s42003-023-05408-7 (PMC10560205; doi:10.1038/s42003-023-05408-7)
Supplement: Supplementary file 3 — Description of Additional Supplementary Files [file 42003_2023_5408_MOESM3_ESM.pdf]

File name: Supplementary table
Description: The supplementary tables 1-3 in the main text.

File name: Supplementary data 1
Description: The supplementary table of the Mendelian randomization analysis results between various cathepsins and lung cancer stratified by smoking behavior

File name: Supplementary data 2
Description: The supplementary table that described the characteristics of genetic variants serving as instrumental variables.

File name: Supplementary data 3
Description: The source data used for generating Figures 1-4 in the main text.
